# A diver’s dilemma – a case report on bronchopulmonary sequestration

**DOI:** 10.1186/s12890-020-1159-1

**Published:** 2020-05-04

**Authors:** Timothy Xin Zhong Tan, Andrew Yunkai Li, James Jie Sng, Mark Lim, Zhi Xiang Tan, Hope Xian’en Ang, Boon Hor Ho, David Zhiwei Law, Anne Ann Ling Hsu

**Affiliations:** 1 Navy Medical Service, Republic of Singapore Navy, 126 Tanah Merah Coast Road, Singapore, 498822 Singapore; 2 Division of Respiratory and Critical Care Medicine, Department of Medicine, National University Hospital, Singapore, Singapore; 3 Department of Respiratory and Critical Care Medicine, Singapore General Hospital, Singapore, Singapore

**Keywords:** Intralobar bronchopulmonary sequestration, Diving and hyperbaric medicine, Diving fitness, Pre-diving medical screening

## Abstract

**Background:**

An asymptomatic SCUBA (Self-contained underwater breathing apparatus) diver was discovered to have an intralobar bronchopulmonary sequestration during routine pre-course screening. This is the first reported case of a diver who, having previously completed several recreational and military diving courses, was subsequently diagnosed with a congenital lung condition, possibly contraindicating diving. Presently, there is no available literature providing guidance on the diving fitness of patients with such a condition.

**Case presentation:**

An asymptomatic 26-year-old male diver was nominated to attend an overseas naval diving course. Prior to this, he had been medically certified to participate in, and had successfully completed other military and recreational diving courses. He had also completed several hyperbaric dives up to a depth of 50 m and 45 recreational dives up to a depth of 30 m. He did not have a history of diving-related injuries or complications. He had never smoked and did not have any medical or congenital conditions, specifically recurrent respiratory infections. As part of pre-course screening requirements, a lateral Chest X-ray was performed, which revealed a left lower lobe pulmonary nodule. This was subsequently diagnosed as a cavitatory left lower lobe intralobar bronchopulmonary sequestration on Computed Tomography Thorax. The diver remains asymptomatic and well at the time of writing and has been accepted to participate in another overseas course involving only dry diving in a hyperbaric chamber, with no prerequisites for him to undergo surgery.

**Conclusion:**

Although bronchopulmonary sequestrations lack communication with the tracheobronchial tree, they may still contain pockets of air, even if not radiologically visible. This can be attributed to anomalous connections which link them to other bronchi, lung parenchyma and/or pores of Kohn. As such, there is a higher theoretical risk of pulmonary barotrauma during diving, leading to pneumothorax, pneumomediastinum, or cerebral arterial gas embolism. Taking these into consideration, the current clinical consensus is that bronchopulmonary sequestrations and all other air-containing lung parenchymal lesions should be regarded as contraindications to diving. Patients who have undergone definitive and uncomplicated surgical resection may be considered fit to dive.

## Background

As recreational SCUBA (Self-contained underwater breathing apparatus) diving continues to gain popularity [1], physicians may be increasingly requested to perform dive medical screenings, as well as manage diving-related emergencies. It is therefore essential that comprehensive diving guidelines, including prerequisites for fitness to dive and diving contraindications, be made easily assessible for reference. It is also critical that physicians be cognisant of certain absolute pulmonary contraindications to diving such as emphysema with bullae and lung cysts, due to the risk of life-threatening complications such as arterial gas embolism.

Bronchopulmonary sequestrations (BPS) constitute 0.15–6.4% of all congenital pulmonary malformations [1], out of which 75% are intralobar sequestrations (ILS) and 25% are extralobar sequestrations (ELS) [2]. They consist of a non-functioning lung unit most commonly found in the left lower lobe (56%), with airway and alveolar elements receiving blood supply from the systemic blood system - most commonly the descending thoracic aorta - and draining mostly to the left atrium in ILS, and the right atrium or azygous vein in ELS [3].

There is presently no literature or guidelines governing the fitness to dive for individuals with BPS. This is the first reported case of a SCUBA diver having previously cleared pre-diving medical screening, and completed several military and recreational diving courses, to be subsequently diagnosed with a congenital lung condition which is a potential contraindication to diving. Expert opinion on the fitness to dive for such individuals is also presented in this case report.

## Case presentation

### Medical history

An asymptomatic 26-year-old male diver was nominated to attend an overseas naval diving course. Prior to this, he had been medically certified to participate in, and had successfully completed other military and recreational diving courses. He had also completed several hyperbaric bounce dives up to a depth of 50 m (~ 164 ft) and 45 recreational dives up to a depth of 30 m (~ 98 ft). He had been well and did not have a history of diving-related injuries or complications such as decompression illness or barotrauma. He had never smoked and did not have any known medical or congenital conditions, specifically recurrent respiratory infections. There was no past surgical history, or family history of respiratory or congenital conditions.

### Investigations

**Fig. 1:**
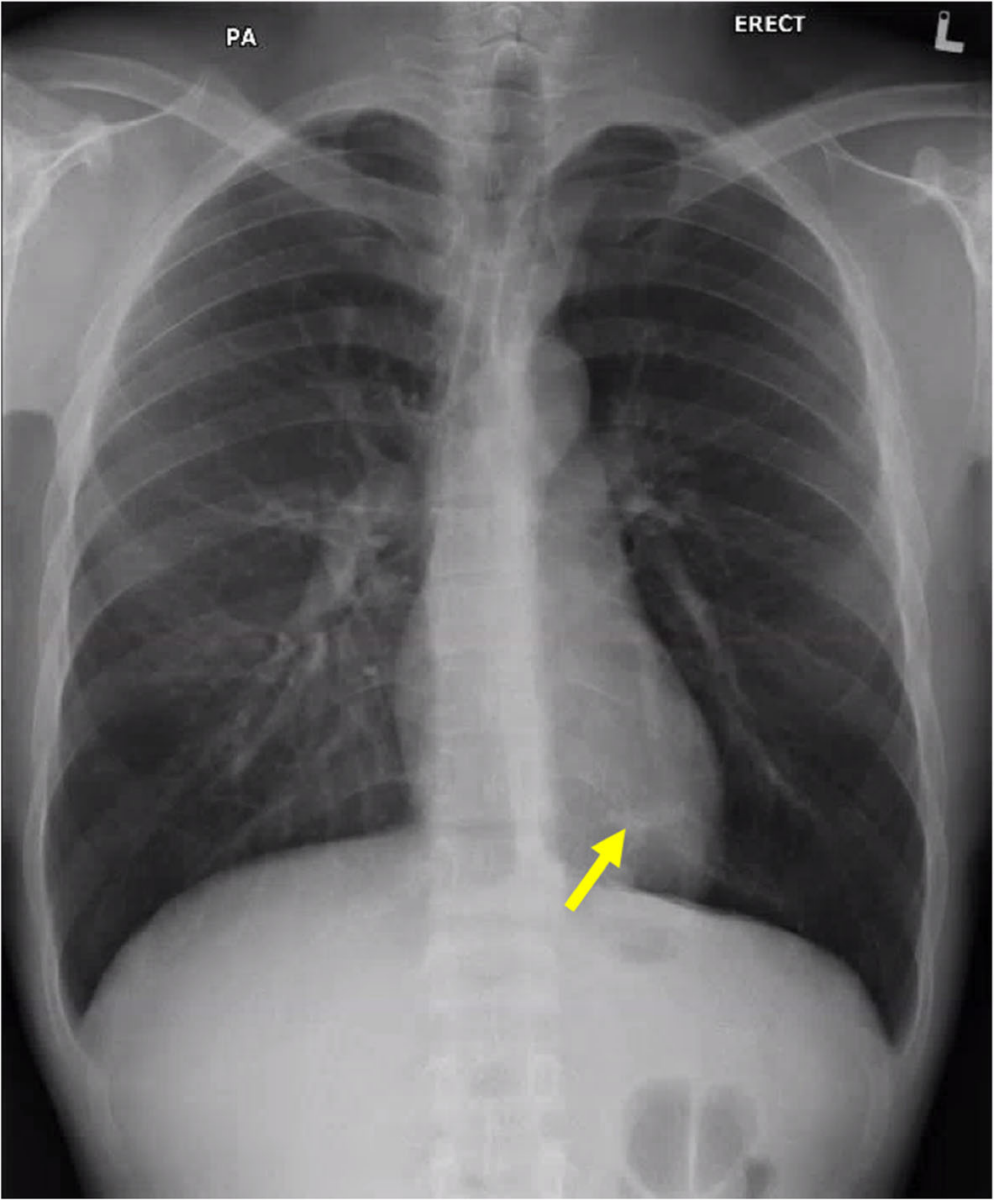
Posteroanterior Chest X-ray revealing a left lower zone nodule adjacent to the vertebral body (yellow arrow)

**Fig. 2:**
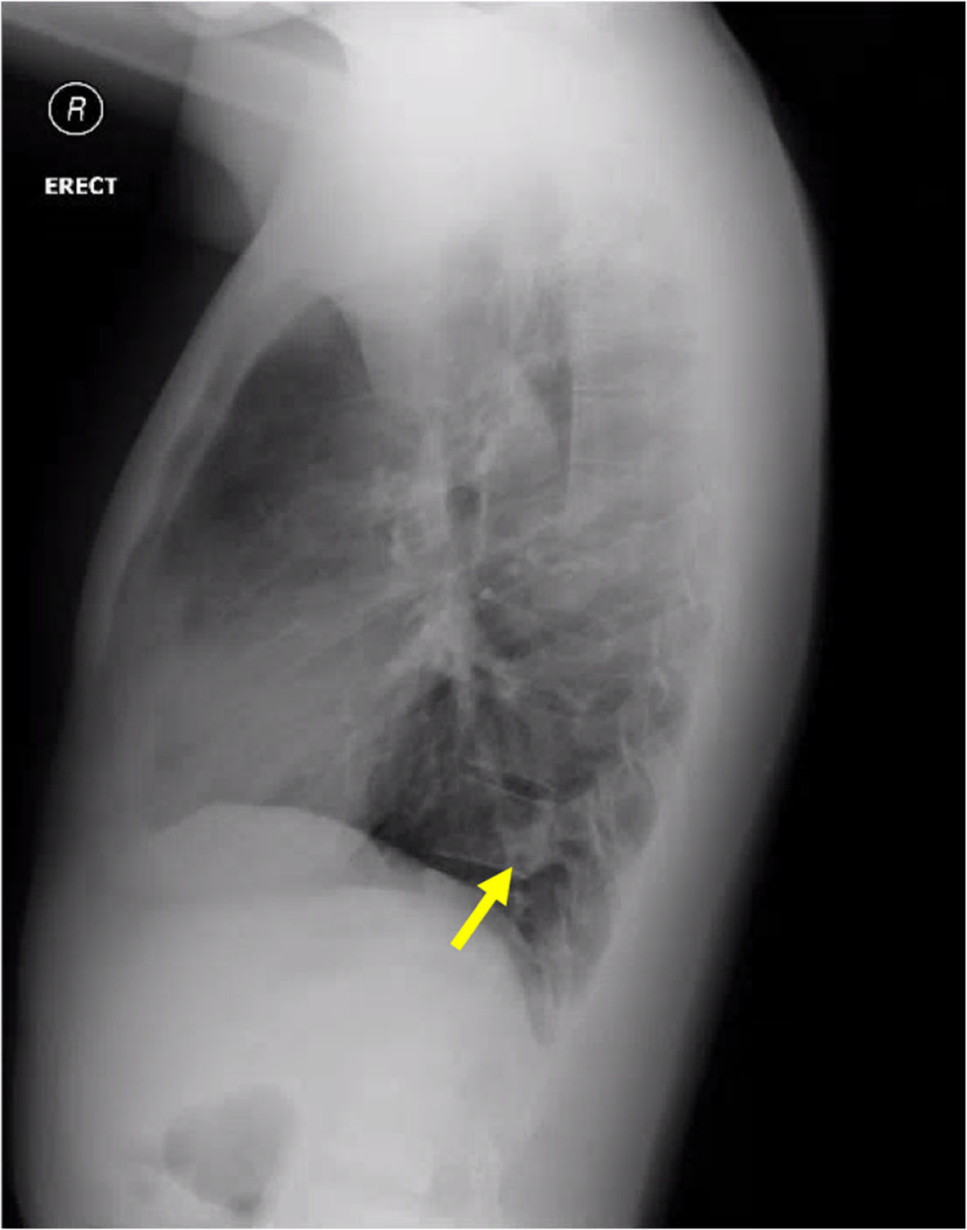
Lateral Chest X-ray more clearly revealing the left lower lobe pulmonary nodule (yellow arrow)

**Fig. 3:**
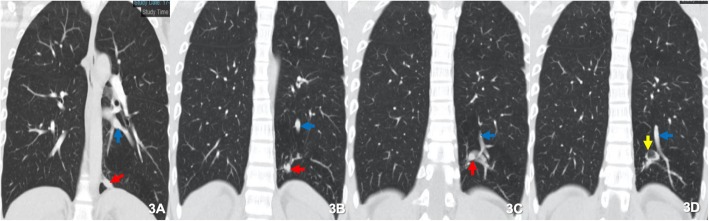
Computed Tomography Pulmonary Angiogram revealing an intralobar pulmonary sequestration which is adjacent to the normal lung without a separate pleura. The blood supply arises from the systemic circulation via the thoracic aorta (red arrows) with drainage into the inferior pulmonary veins (blue arrows). A 1.2 cm cavitation is seen (yellow arrow). (140 kV, Lung window center − 600, width 1500)


As part of pre-course screening requirements for his overseas course, a physical examination, blood tests, as well as posteroanterior (PA) and lateral Chest X-rays (CXR) were performed (Figs. 1 and 2). The physical examination findings were unremarkable. The results of the blood tests were also noted to be normal. However, the lateral CXR revealed a left lower lobe pulmonary nodule adjacent to the vertebral body. This was not reported on his previous PA-only CXRs taken in 2011, 2012, and 2018. A low dose Computed Tomography (CT) Thorax was performed, which revealed a 3 cm serpiginous lesion in the left lower lobe. A CT Pulmonary Angiography which was subsequently performed to rule out any pulmonary arteriovenous malformation, revealed a 9.7 × 9.1 mm left lower lobe intralobar bronchopulmonary sequestration (ILS) instead. This comprised a cavity, receiving a direct arterial blood supply from the descending aorta and draining into the inferior left pulmonary vein (Fig. 3, Video 1). There were no other abnormalities noted. As the lesion was noted to be small, and the patient was asymptomatic and well, functional tests such as a lung function test were not done. The patient was referred to a pulmonologist for evaluation on fitness to dive, as well as for further management of his lung lesion.

**Additional file 1: Video 1.** Computed Tomography Pulmonary Angiogram, Coronal Lung Window. A Computed Tomography Pulmonary Angiogram was performed which revealed a 9.7 × 9.1 mm left lower lobe intralobar bronchopulmonary sequestration with a cavity and direct aortic supply from the descending aorta and venous drainage into the inferior left pulmonary vein. The arterial and venous vessels are highlighted in the video (red and blue arrow respectively).


### Outcomes

The diver remains asymptomatic and well at the time of writing this case report and has been accepted to participate in another overseas course involving only dry diving in a hyperbaric chamber (with immediate access to a resuscitation trolley and a pleural catheter set). There are no course prerequisites for him to undergo surgery.

## Discussion

This is the first reported case of a SCUBA diver who, having previously completed several recreational and military diving courses, was subsequently diagnosed with a congenital lung condition, possibly contraindicating diving. Identification of a systemic arterial supply via CT Pulmonary Angiography is pathognomonic, aiding diagnosis by facilitating differentiation of BPS from other congenital pulmonary diseases, and also subsequently guiding surgical management. Leoncini G et al. previously reported a case of a 29-year-old Italian man with ELS, who presented with haemoptysis and was treated with embolization. He was subsequently certified with a license to dive, but there is no documentation of his diving history prior to, and after diagnosis and treatment of his ELS [4].

Extralobar sequestrations are separated from the normal lung by its own pleura, have a 3:1 male predominance, and are more likely to be associated with other congenital anomalies: 43% of all ELS cases compared to 17% of ILS cases [1]. They are therefore usually detected much earlier, either via routine prenatal ultrasound screens, or after patients present symptomatically [5]. Conversely, ILS are located within a normal lobe of the lung, do not have a gender predominance, and are associated with recurrent lung infections due to the increased likelihood of associated anomalous bronchial communications. These cases are therefore usually diagnosed later in life, about 50% after the age of 20 [5]. On plain chest radiographs, such bronchopulmonary sequestrations may present as a non-specific cystic mass in the lung base with air-fluid levels documented in 26% of cases [2]. Infective changes may also be seen if these lesions are actively infected or have previously been infected [1, 2].

### Pre-diving screening and assessment

Pre-diving medical screening for military and recreational diving courses vary widely across countries and various organizations. Some diving courses require candidates to undergo PA and lateral CXRs, or even CT thorax examinations, while others, particularly recreational diving courses, will not require a PA CXR unless medically indicated (e.g. elderly, heavy smoker, symptomatic patients) [6–8]. Although this case study appears to suggest that a lateral CXR should be performed for all pre-diving medical screening, its utility is marginal and should be weighed against other issues such as higher false positive rates, increased radiation exposure (0.02 vs 0.08 mSv for PA and lateral CXR respectively), and additional costs [1]. Advances in CT Thorax and other higher imaging modalities also preclude the use of the lateral CXR. Regardless, the adoption and administration of more uniform pre-diving medical screening standards should be considered.

### Pulmonary considerations and contraindications to SCUBA diving

SCUBA diving affords humankind the ability to explore the underwater realm. However, exposure to the hyperbaric environment poses unique demands on human physiology. Boyle’s law dictates that volume is inversely proportional to absolute pressure exerted on the body. As such, as pressure increases at deeper depths underwater, there is a reduction in the volume of gas-containing spaces in the body, and vice-versa. If these gas-containing spaces do not equalise with ambient pressure, tissue injury can result from the pressure differences on descent and lung barotrauma can result from overexpansion on ascent, resulting in life threatening complications such as pneumothorax, pneumomediastinum and arterial gas embolism [9].

In view of these considerations, many international diving and hyperbaric medical societies such as the Undersea and Hyperbaric Medical Society [10] and the South Pacific Underwater Medicine Society [11] stipulate that pulmonary conditions such as emphysema with bullae and lung cysts which predispose patients to abnormal gas-containing spaces pose an absolute contraindication to diving. Other absolute pulmonary contraindications include all acute pulmonary disorders, and poorly controlled asthma, while relative pulmonary contraindications include well-controlled asthma (FEV1 and peak-flow values above 80% of expected values, normal FEV1/FVC and negative exercise tests), chronic bronchitis, and spontaneous pneumothoraces treated with bilateral surgical pleurectomies [1]. These risks are understandably higher for wet/SCUBA diving as opposed to dry/hyperbaric diving, with the challenging operating environment in the former resulting in increased demands in terms of buoyancy control, physical exertion and the added risk of drowning in the event of mishaps. Pulmonary arterio-venous malformations should also be considered an absolute contraindication to diving as they act as a right-to-left shunt, exposing divers to increased risks of decompression illness through paradoxical gas embolism [12].

The current recommended management of BPS is contingent on two main factors – [1] the presence of symptoms, and/or [2] the presence of complications such as infection. The standard of care for symptomatic and/or complicated cases is surgical resection, usually in the form of an open or thoracoscopic lobectomy or segmentectomy [13], although arterial coil embolization has also been documented [4, 5, 13]. While there has yet to be a recorded mortality secondary to operative management of BPS, the post-operative complication rate is known to be 5–28%, with complications such as pneumonia, chyle leak, and recurrent laryngeal nerve injury reported in the literature [4, 7, 13, 14]. There is currently no consensus on the management of asymptomatic and uncomplicated cases [3, 14]. The argument for surgery for all cases of BPS is that symptoms and complications such as infections and malignant degeneration may develop later in life, rendering emergent surgery potentially life-threatening and an increased risk of post-operative complications [15]. However, observation may be considered if the lesion is small, non-cystic, and consistent with the findings of ELS [15], and surgical intervention indicated only for larger-sized lesions (mean diameter, 5.8 cm) [14]. If conservative management is adopted, regular reviews are recommended [11, 15], although at present there is no consensus on the modality, frequency or duration of such follow-up.

Although BPS lacks communication with the tracheobronchial tree [1, 5], they may still contain non-communicating pockets of air, even if not radiologically visualised. These air pockets result from anomalous connections linking BPS to other bronchi or lung parenchyma [2] and/or pores of Kohn, which are physiological collaterals with adjacent alveoli [16]. Our patient was noted to have a cavitary ILS draining into the systemic circulation via the pulmonary vein, which leads directly to the heart. Thus, he is at a higher theoretical risk of lung barotrauma if exposed to significant changes in pressure, possibly leading to life threatening conditions such as pneumothorax and cerebral arterial gas embolism. In view of these potentially life-threatening complications, the current clinical consensus in our local setting is that BPS and all other air-containing lung parenchymal lesions should be regarded as an absolute contraindication to diving, especially if pockets of air or air-fluid levels are visualised on radio-imaging, unless definitive uncomplicated surgical resection is performed. If clinically required, surgical resection is the preferred modality of treatment as opposed to embolization, as complete embolisation does not necessarily provide the assurance of complete involution of the sequestration. And even if it does, this process is likely to take months to years [4, 5]. To the best of our knowledge, there are also no prior documented cases of embolisation performed for BPS with radiologically visible air pockets [17]. Furthermore, it is difficult to ascertain if the air present in such cases are secondary to previous infection, which may be considered a contraindication to embolisation as retained nonaerated pulmonary parenchymal tissue may form a nidus for infection [5]. Lastly, although resection should not significantly compromise lung function if done early in life or if the lesion is small [10], pre- and post-operative lung function tests should be performed to assess for adequacy prior to diving.

As bronchopulmonary sequestrations (BPS) are rare and often retrocardiac in location, a high index of suspicion and radiological experience is essential to aid definitive diagnosis, which may be made with the use of adjunctive radiological tests such as lateral CXR, CT Thorax and CT Pulmonary Angiography. Identification of a systemic arterial supply is pathognomonic and differentiates BPS from other congenital pulmonary diseases. Bronchopulmonary sequestrations and all air-containing lung parenchymal lesions should be regarded as absolute contraindications to SCUBA diving, unless definitive uncomplicated surgical resection is performed.

## Data Availability

The data used during this study are included in this article and are also available from the corresponding author on reasonable request. The authors warrant that the article is original, is not under consideration by another journal, and has not been published previously. Preliminary data was not presented previously at any conference. The undersigned authors agree to transfer all copyright ownership of the manuscript to BMJ Pulmonary Medicine and BioMed Central in the event the work is published. The paper will not be published elsewhere in the same form, in English or in any other language, including electronically, without the written consent of the copyright holder.

## Abbreviations

SCUBA: Self-contained underwater breathing apparatus
BPS: Bronchopulmonary sequestrations
ILS: Intralobar sequestrations
ELS: Extralobar sequestrations
CXR: Chest X-ray
PA: Posteroanterior
CT: Computed Tomography
FEV1: Forced expiratory volume in 1 s
FVC: Forced vital capacity

## References

[1] Rusoke-Dierich O. Assessment for Diving Fitness for Recreational Divers. In *Diving Medicine*. Springer International Publishing; 2018. p. 360–3..

[2] Ko SF, Ng SG, Lee TY, Wan YL, Liang CD, Lin JW, et al. Noninvasive imaging of bronchopulmonary sequestration. AJR Am J Roentgenol. 2000. 10.2214/ajr.175.4.1751005.. 10.2214/ajr.175.4.1751005 11000154

[3] Alsumrain M, Ryu JH. Pulmonary sequestration in adults: a retrospective review of resected and unresected cases. BMC Pulm Med. 2018. 10.1186/s12890-018-0663-z.. 10.1186/s12890-018-0663-z PMC5989460 29871620

[4] Leoncini G, Rossi UG, Ferro C, Chessa L. Endovascular treatment of pulmonary sequestration in adults using Amplatzer® vascular plugs. Interact Cardiovasc Thorac Surg. 2011. 10.1510/icvts.2010.246546.. 10.1510/icvts.2010.246546 20940163

[5] Ellis J, Brahmbhatt S, Desmond D, Ching B, Hostler J. Coil embolisation of intralobar pulmonary sequestration – an alternative to surgery: a case report. J Med Case Rep. 2018. 10.1186/s13256-018-1915-5.. 10.1186/s13256-018-1915-5 PMC6302303 30572944

[6] European Diving Technology Committee. Fitness to dive standards: guidelines for medical assessment of working divers. 2003. http://www.edtc.org/EDTC-Fitnesstodivestandard-2003.pdf. Accessed 22 Jun 2019..

[7] Ministry of Labour, Ontario. Code for the medical examination of divers. 2014. https://www.labour.gov.on.ca/english/hs/pubs/medcode_diving.php. Accessed 10 Nov 2019..

[8] American Academy of Underwater Sciences. AAUS standards for scientific diving. 2019. https://www.aaus.org/AAUS/About/Diving_Standards/AAUS/Diving_Standards.aspx?hkey=25acfc9a-aea5-4e7f-86c6-9c514c1e764c. Accessed 14 Nov 2019..

[9] Bove AA. Diving medicine. Am J Respir Crit Care Med. 2014. 10.1164/rccm.201309-1662CI.. 10.1164/rccm.201309-1662CI 24869752

[10] Recreational Scuba Training Council. Medical Statement. 2007. http://wrstc.com/downloads/10%20-%20Medical%20Guidelines.pdf. Accessed 27 Mar 2020..

[11] South Pacific Underwater Medicine Society. Guidelines on Medical Risk Assessment for Recreational Diving. https://www.spums.org.au/content/spums-full-medical-0. Accessed 27 Mar 2020.. 10.28920/dhm50.3.273-277 PMC7819720 32957130

[12] Shovlin CL, Wilmshurst P, Jackson JE. Pulmonary arteriovenous malformations and other pulmonary aspects of hereditary haemorrhagic telangiectasia. Eur Respir Monogr. 2011. 10.1183/1025448x.10008410..

[13] Singh R, Davenport M. The argument for operative approach to asymptomatic lung lesions. Semin Pediatr Surg. 2015. 10.1053/j.sempedsurg.2015.02.003.. 10.1053/j.sempedsurg.2015.02.003 26051052

[14] Criss CN, Musili N, Matusko N, Baker S, Geiger JD, Kunisaki SM. Asymptomatic congenital lung malformations: is nonoperative management a viable alternative? J Pediatr Surg. 2018. 10.1016/j.jpedsurg.2018.02.065.. 10.1016/j.jpedsurg.2018.02.065 29576400

[15] Stanton M. The argument for a non-operative approach to asymptomatic lung lesions. Semin Pediatr Surg. 2015. 10.1053/j.sempedsurg.2015.01.014.. 10.1053/j.sempedsurg.2015.01.014 26051051

[16] Oldham MJ, Moss OR. Pores of Kohn: forgotten alveolar structures and potential source of aerosols in exhaled breath. J Breath Res. 2019. 10.1088/1752-7163/ab0524.. 10.1088/1752-7163/ab0524 30731449

[17] Cui L, Xu XR, Chui JG, Fu ZJ, Bi YM, Wang JC. A fighter pilot case of pulmonary sequestration. Aviat Space Environ Med. 2012. 10.3357/asem.3261.2012.. 10.3357/asem.3261.2012 23316546

